# Controllable and Uncontrollable Stress Differentially Impact Fear Conditioned Alterations in Sleep and Neuroimmune Signaling in Mice

**DOI:** 10.3390/life12091320

**Published:** 2022-08-26

**Authors:** Austin M. Adkins, Laurie L. Wellman, Larry D. Sanford

**Affiliations:** Sleep Research Laboratory, Center for Integrative Neuroscience and Inflammatory Diseases, Department of Pathology and Anatomy, Eastern Virginia Medical School, Norfolk, VA 23507, USA

**Keywords:** stressor controllability, sleep, neuroinflammation, fear memory, extinction learning

## Abstract

Stress induces neuroinflammation and disrupts sleep, which together can promote a number of stress-related disorders. Fear memories associated with stress can resurface and reproduce symptoms. Our previous studies have demonstrated sleep outcomes can be modified by stressor controllability following stress and fear memory recall. However, it is unknown how stressor controllability alters neuroinflammatory signaling and its association with sleep following fear memory recall. Mice were implanted with telemetry transmitters and experienced escapable or inescapable footshock and then were re-exposed to the shuttlebox context one week later. Gene expression was assessed with Nanostring^®^ panels using RNA extracted from the basolateral amygdala and hippocampus. Freezing and temperature were examined as behavioral measures of fear. Increased sleep after escapable stress was associated with a down-regulation in neuro-inflammatory and neuro-degenerative related genes, while decreased sleep after inescapable stress was associated with an up-regulation in these genes. Behavioral measures of fear were virtually identical. Sleep and neuroimmune responses appear to be integrated during fear conditioning and reproduced by fear memory recall. The established roles of disrupted sleep and neuroinflammation in stress-related disorders indicate that these differences may serve as informative indices of how fear memory can lead to psychopathology.

## 1. Introduction

Fear memories are normal components of stress-related learning [1,2]; however, when improperly processed, they can be recalled in response to non-threatening environmental contexts or stimuli [3,4,5,6], prompting inappropriate fear reactions [7,8,9,10,11]. These maladaptive responses within fear neurocircuitry are implicated in fear- and anxiety-based psychiatric disorders [3,12,13,14,15] and are thought to involve abnormal fear extinction following a stressful event [12,13,14,15] that lead to the persistent and inappropriate recall of fear memories [3,11,16]. The continued intrusion of fear memories activates stress response mechanisms, and can increase neuroinflammation [17,18,19,20], which is thought to play a negative role in fear memory extinction and lead to the development of posttraumatic stress disorder (PTSD), and mood and anxiety disorders in humans [12,14,15].

Sleep disturbances both before and after significant stress are implicated in stress-related pathology [21,22,23,24,25,26] and are defining features of PTSD [23,27,28,29,30]. Disturbed sleep may impact multiple systems; it is thought to be important for memory consolidation [31,32,33,34,35,36], emotional processing [37,38], and it has reciprocal influences with the immune system [39,40,41,42,43,44]. Stress-induced sleep loss can alter immune signaling and contribute to a heightened inflammatory response [17,45,46,47].

While stress is typically associated with negative health outcomes, animal studies have repeatedly shown that outcomes can vary depending on whether the stressor was controllable or not [48,49,50,51,52,53], including differences in sleep [25,54]. Mice trained with controllable stress (modeled by escapable shock (ES)) show increases in rapid eye movement (REM) sleep, whereas mice trained with uncontrollable stress (modeled by inescapable shock (IS)) show decreases [25,54]; subsequent fear-memories associated with ES and IS produce similar alterations in sleep [55].

How memories of controllable and uncontrollable stress impact the neuroimmune response, and its relationship to sleep, are not known. The goal of this study was to assess how stressor controllability can influence the formation of fear memories, and whether fear memories associated with controllable and uncontrollable stress differentially alter subsequent neuroinflammatory, sleep, and behavioral responses. We hypothesize that increased sleep associated with ES after fear memory recall will be associated with protective effects in the immune response, whereas decreased sleep associated with IS will not.

In this study, we investigated the effects of fear memories associated with ES and IS on sleep and on the neuroimmune response in the basolateral nucleus of the amygdala (BLA) and hippocampus (HPC), using our established ES and IS paradigm [25]. To conduct the study, mice were implanted intraperitoneally with telemetry transmitters for recording EEG activity, gross body activity, and whole-body temperature. They were exposed to the ES-IS paradigm for two consecutive days, then re-exposed to the shock context alone (CTX) one week later for assessment of fear memory recall. Sleep was recorded following each training day and after CTX. Then, regions of interest (BLA and HPC) were micro-dissected, RNA isolated, and inflammatory gene expression levels measured via mRNA levels. We also examined freezing as an index of behavioral fear, stress-induced hyperthermia (SIH) as an index of the stress response, and changes in sleep.

## 2. Materials and Methods

### 2.1. Subjects

Twenty-seven male C57BL/6 mice (n = 5–8 per group) approximately 8 wks of age and weighing 25–30 g were obtained from Charles River Laboratories (Wilmington, MA, USA) and individually housed. Food and water were available ad libitum and nestlets were provided as enrichment. Housing and recording rooms were kept on a 12:12 light:dark cycle. Ambient temperature was maintained at 24.5 °C ± 0.5 °C. All procedures were conducted in accordance with the National Institutes of Health Guide for the Care and Use of Experimental Animals and were approved by Eastern Virginia Medical School’s Institutional Animal Care and Use Committee (Protocol#: 17-015).

### 2.2. Surgery

Following a 2-week habituation period, all mice were implanted intraperitoneally with telemetry transmitters (ETA F10 or F20, Data Sciences International; Minneapolis, MN) for recording EEG activity, gross body activity, and whole-body temperature. Leads from the transmitter body were led subcutaneously to the head, and the free ends were placed into holes drilled in the dorsal skull to allow for recording. All surgeries were conducted under isoflurane (inhalant: 5% induction; 2% maintenance) anesthesia. Ibuprofen (30 mg/kg, oral) was continuously available in each animal’s drinking water for 24–48 h pre-operatively and for a minimum of 72 h post-operatively for pain relief. All animals received prophylactic potassium penicillin (25 IU/g), gentamicin (0.005 mg/g), and dexamethasone (0.0005 mg/g) subcutaneously on the day of surgery.

### 2.3. Training Procedures

After a post-surgery recovery period of 4 weeks, a baseline sleep recording was obtained. Mice were then randomly assigned to an experimental group by matched weights not linked to any subject identifiers. On experimental days 1 and 2, mice were shock trained (ST) with escapable (ES) or inescapable (IS) footshock (20 footshocks, 0.5 mA, 5.0 s max. duration, 1 min inter-trial intervals) in a shuttlebox. The ES group had the ability to learn they could behaviorally terminate the footshock by moving to the opposite side of the shuttlebox chamber; the yoked IS group could not control the shock. Termination of shock for an ES mouse also terminated the shock to its yoked IS mouse in a separate shock chamber (Coulbourn Instruments, Model E10-15SC), ensuring each yoked set of mice received the same duration of shock. On day 7, mice were re-exposed to the shuttlebox but did not receive footshocks (CTX). A mock-trained (MT) control group was exposed to the shuttlebox on each experimental day but not shocked. A home cage (HC) control group remained in their cage for the entirety of the study. Recording occurred over a 20 h period at baseline (before training) and immediately after each ST day, and over a 2 h period after CTX. All training took place during the light period in the 3rd h, and sleep recording took place during the 4th h, after lights on.

### 2.4. Data Recording and Determination of Sleep State

Sleep recording occurred within the same room the animals were housed. For recording, individual cages were placed on a telemetry receiver (Model RPC-1, Data Sciences International; Minneapolis, MN, USA), and the transmitter activated with a magnet. When the animals were not on study, the transmitter was inactive. Telemetry signals were processed and collected using DataquestART software (sampling rate of 256 Hz, Version 4.0, Data Sciences International; Minneapolis, MN, USA), then visually scored by a trained individual blinded to treatment condition in 10 s epochs using the SleepSign sleep analysis program (Kissei Comtec Co.; Tokyo, Japan). Epochs were scored as either active wakefulness (AW), quiet wakefulness (QW), non-rapid eye movement (NREM) sleep, or rapid eye movement (REM) sleep based on EEG and gross whole-body activity. Active wakefulness was scored based on the presence of high frequency, low amplitude EEG, and body activity. Quiet wakefulness was scored based on the presence of high frequency, low amplitude EEG, and body inactivity. NREM was scored by the presence of low frequency, high amplitude EEG interspersed with spindles and body inactivity. REM was scored by the presence of high frequency, low amplitude EEG with the presence of theta rhythms and body inactivity.

The scored data for sleep parameters during baseline and ST recordings were analyzed for the first 2 h of the recording period, the total 8 h light period, the total 12 h dark period, and across the total 20 h recording period. The data were analyzed with Group (ES and IS or HC and MT) × Treatment (Baseline, ST 1, and ST 2) mixed-factor analyses of variance (ANOVA) with repeated measures on Treatment. Post hoc comparisons following significant ANOVAs were performed using Tukey’s test. Statistical methods were generated using PRISM GraphPad software (version 9.4.0). The following parameters were evaluated: total REM sleep, and total NREM sleep.

The scored data for sleep parameters following CTX were analyzed for the 2 h recording period. The data were analyzed with Group (ES and IS or HC and MT) × Treatment (Baseline, ST 1, ST 2, and CTX) mixed-factor ANOVA with repeated measures on Treatment as described above. The parameters listed above were also evaluated.

### 2.5. Determination of Freezing

ST and CTX sessions were videotaped (KT&C analog mini camera, model KPC-S400P1, 420 TVL) for visual scoring of freezing, defined as the absence of body movement except for respiration [56,57]. A trained individual visually scored freezing using VLC Media Player (Version 3.0.16, played back at 29.97 frames per s) in 5 s intervals over the course of the 30 min the mice were in the shuttlebox during ST 1, ST 2, and CTX. The percentage of time spent freezing was calculated as FT%: freezing time/observed time × 100.

### 2.6. RNA Extraction

All groups were euthanized 2 h following CTX (immediately after sleep recording) via isoflurane sedation (inhalant: 5%, ≤5 min duration) and perfused with PBS for assessment of inflammatory gene expression. Brains were extracted and regions of interest (BLA and HPC) micro-dissected, snap frozen and stored in RNAlater (ThermoFisher Scientific, Waltham, MA, USA) at −80 °C until analysis. RNA was isolated using the Qiagen RNeasy Mini Kit. Samples from HPC were loaded into the NanoString Mouse Neuroinflammation (NI) panel and samples from BLA were loaded into the NanoString Mouse Alzheimer’s Disease (AD) panel. Each panel contains a set of over 770 pre-selected genes related to neuroinflammatory and immune processes. Results from the panels were uploaded to the nSolver database (Version 4.0.70; NanoString Technologies; Seattle, WA, USA) to assess relative levels of neuroinflammatory markers within the NI and AD panels. Gene expression and pathway profiles were compiled for each group to assess expression levels relative to the fear response following CTX.

Data were normalized to the internal positive and negative controls to account for slight differences in assay efficiencies. The normalized gene counts for each gene in each assay were then divided by the appropriate normalization factor and averaged for the samples of each mRNA type to generate counts normalized to the internal reference genes. Fold changes in gene transcript levels were determined relative to basal levels detected in the HC group. Relative fold changes in transcript levels for each determined gene were compared between groups. The data were analyzed within nSolver using multiple *t*-tests with Benjamini-Yekutieli correction on Group and related genes for each gene of each panel. The following parameters were evaluated: differences in ES compared to HC, differences in IS compared to HC, differences in MT compared to HC, and differences between ES and IS.

## 3. Results

### 3.1. REM Sleep

Baseline REM did not significantly differ between any of the 4 experimental groups for any parameter examined and did not significantly differ between the MT and HC groups for any parameter examined on any experimental day (Supplementary Figure S1A,B). There also were no significant differences in REM amounts between ES and the HC and MT control groups for any parameter examined. However, the IS group had significantly less REM sleep on ST 1, ST 2, and CTX days compared to the HC and MT control groups. Furthermore, compared to IS mice, ES mice showed increases in REM sleep following ST 1, ST 2, and CTX demonstrating the effect of stressor controllability on footshock- and fear-induced alterations in REM (Table 1). Additionally, the IS group had the lowest percentage of REM compared to any other group during ST 1, ST 2, and CTX (Supplementary Table S1). Further comparisons below focus only on comparisons between the ES and IS treatment groups.

**Figure 1:**
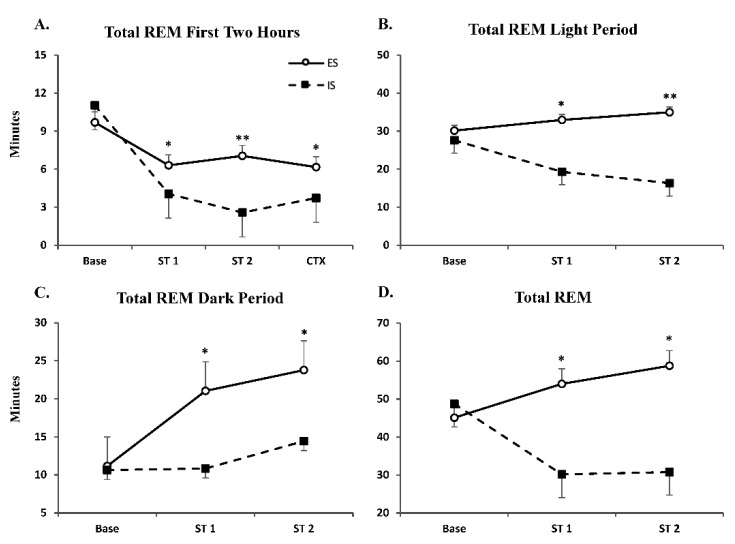
**Rapid Eye Movement Sleep is Reduced After Inescapable but not Escapable Stress.** Total REM ± SEM duration plotted following Baseline (Base), shock training days (ST 1 and ST 2), and context re-exposure (CTX) during the (**A**) first 2 h, (**B**) light period (8 h), (**C**) dark period (12 h) and (**D**) total 20 h. Significant differences in ES vs. IS: * *p* < 0.05, ** *p* < 0.01.

Analysis of total REM over a 2 h recording period (Figure 1A) revealed a significant main effect for group (F_1,55_ = 6.16; *p* < 0.01) and a significant group x treatment interaction (F_3,55_ = 14.90; *p* < 0.00001); Tukey’s post hoc test revealed a significant difference between groups for ST 1, ST 2, and CTX (*p* < 0.05, *p* < 0.01, *p* = 0.02, respectively). Compared to IS mice, ES mice exhibited significantly more REM following ST 1, ST 2, and CTX. Analysis of REM over the 8 h recording during the light period (Figure 1B) revealed a significant main effect for group (F_1,35_ = 9.23; *p* < 0.01); Tukey’s post hoc test revealed a significant difference between groups for ST 1 and ST 2 (*p* < 0.05, *p* = 0.01, respectively). Compared to IS mice, ES mice exhibited significantly greater REM following ST 1 and ST 2. Analysis of REM over the 12 h recording during the dark period (Figure 1C) revealed a significant main effect for group (F_1,35_ = 7.36; *p* < 0.01); Tukey’s post hoc test revealed a significant difference between groups for ST 1 and ST 2 (*p* < 0.05, *p* = 0.05, respectively). Compared to IS mice, ES mice exhibited significantly greater REM during baseline and following ST 1 and ST 2. Analysis of REM over the total 20 h recording period (Figure 1D) revealed a significant main effect for group (F_1,35_ = 10.15; *p* < 0.01); Tukey’s post hoc test revealed a significant difference between groups for ST 1 and ST 2 (*p* < 0.05, *p* < 0.05, respectively). Compared to IS mice, ES mice exhibited significantly greater REM following ST 1 and ST 2.

### 3.2. NREM Sleep

Baseline NREM did not significantly differ between any experimental groups for any parameter examined. NREM amounts did not significantly differ between MT and HC groups on any experimental day for any parameter examined (Supplementary Figure S1C,D). NREM amounts in the HC and MT control groups also did not significantly differ from ES or IS treatment groups on any experimental day (Table 2). However, ES showed increases in NREM sleep following ST 1 and ST 2 compared to IS (Table 2). Given that the only significant differences in NREM sleep occurred between the ES and IS groups, further comparisons focused on ES and IS treatment groups.

**Figure 2:**
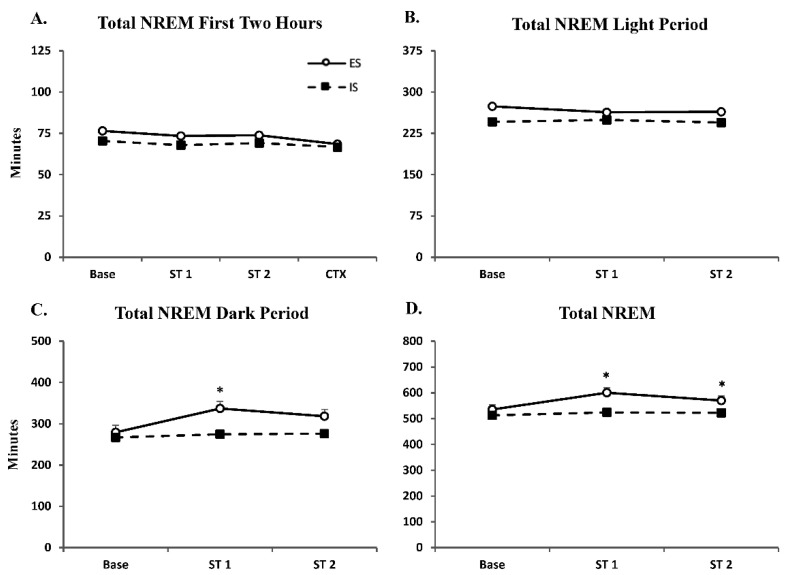
**Non-Rapid Eye Movement Sleep is Increased After Escapable Stress.** Total NREM ± SEM duration plotted following Baseline (Base), shock training days (ST 1 and ST 2) and context re-exposure (CTX) during the (**A**) first 2 h, (**B**) light period (8 h), (**C**) dark period (12 h) and (**D**) total 20 h. Significant differences in ES vs. IS: * *p* < 0.05.

Analysis of total NREM revealed no significant differences over the 2 h recording period (Figure 2A; F_1,47_ = 2.31) and the 8 h recording during the light period (Figure 2B, F_1,35_ = 6.27). Analysis of NREM over the 12 h recording during the dark period (Figure 2C) revealed a significant main effect for group (F_1,35_ = 9.80; *p* < 0.01); Tukey’s post hoc test revealed a significant difference between groups for ST 1 (*p* < 0.05). Compared to IS mice, ES mice exhibited significantly greater NREM following ST 1. Analysis of NREM over the total 20 h recording period (Figure 2D) revealed a significant main effect for group (F_1,35_ = 11.16; *p* < 0.01); Tukey’s post hoc test revealed a significant difference between groups for ST 1 and ST 2 (*p* < 0.05, *p* = 0.05, respectively). Compared to IS mice, ES mice exhibited significantly greater NREM following ST 1 and ST 2.

### 3.3. Freezing and Body Temperature

**Figure 3:**
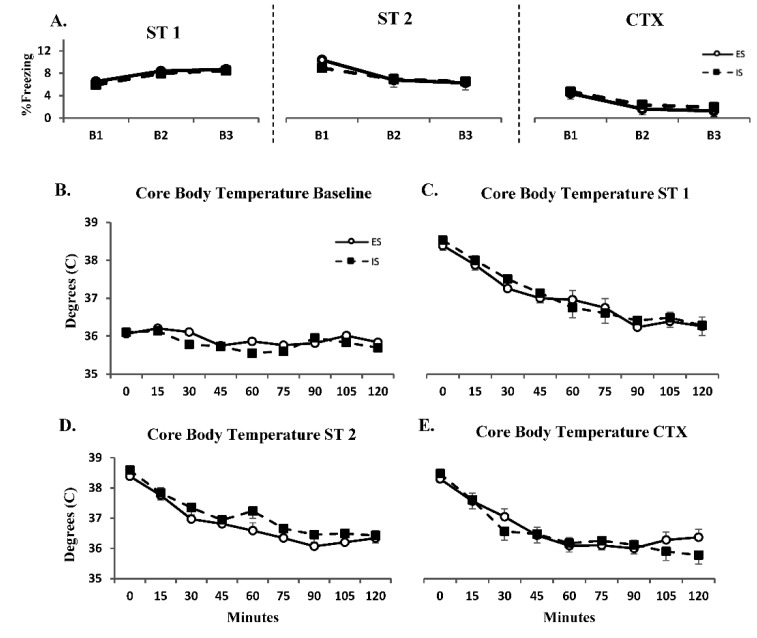
**Freezing Behavior and Stress-Induced Hyperthermia Does Not Differ Between Escapable and Inescapable Stress.** (**A**) Percent time freezing ± SEM plotted in 10 min blocks (B1-3) for the total 30 min timeframe during each shock training day (ST 1 and ST 2) and context re-exposure (CTX). Average core body temperature ± SEM plotted in 15 min intervals for a total of 2 h (**B**) at baseline, (**C**) after shock training day 1 (ST 1), (**D**) after shock training day 2 (ST 2) and (**E**) after context re-exposure (CTX).

Total amount of freezing for each 30 min ST day and CTX did not differ between ES and IS groups or between experimental days (Figure 3A). The MT group exhibited minimal freezing behavior (average of 0.043% total freezing behavior across all experimental days, data not shown).

Core body temperature was examined for a total of 2 h during baseline, following ST 1 and ST 2, and CTX (Figure 3B–E, respectively). Groups did not display a significant difference in body temperature during baseline. Both ES and IS groups displayed similar increases in core body temperature following ST 1, ST 2, and CTX which returned to baseline levels within 2 h. HC and MT groups maintained an average core body temperature of 36.1 °C ± 0.16 throughout the entirety of the study (data not shown).

### 3.4. Neuroinflammation

Analyses of gene markers of neuroinflammation and inflammatory-associated pathways in the HPC revealed significant differences between groups. Overall, ES showed decreases in markers of NI and immune system activation following CTX while IS showed increases in markers of NI and immune system activation. There were minimal differences in gene expression between the MT and HC control groups. MT showed a minor up-regulation of genes regulating cellular stress responses (*Ago4*, *p* < 0.05; *Rac2*, *p* < 0.05); cytokine signaling (*Ccl3*, *p* < 0.05; *Gpr84*, *p* < 0.05; *Tnfrsf25*, *p* < 0.05); and the innate immune response (*Lcn2*, *p* = 0.01) compared to HC. (Supplementary Figure S2A).

**Figure 4:**
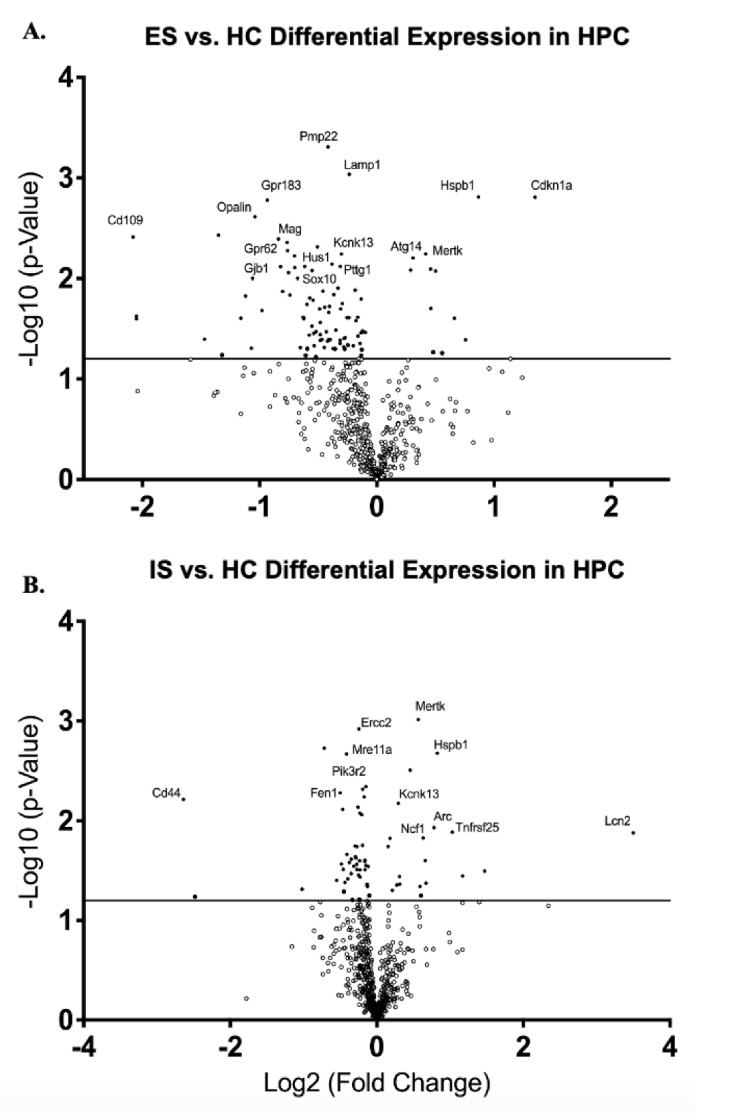
**Escapable Stress Decreases the Expression of Pro-inflammatory Immune Genes.** Volcano plot displaying each gene expression levels in HPC compared to home cage (HC) control following CTX for (**A**) escapable stress (ES) and (**B**) inescapable stress (IS) groups. Statistically significant genes fall above the horizontal line, and highly differentially expressed genes fall to either side of the zero on the *x*-axis. The most relevant genes are labelled in the plot.

Analysis of gene expression via relative mRNA levels within HPC revealed significantly different expression levels between ES and IS groups when compared to HC. ES mice showed a down-regulation of many genes associated with re-myelination (*Mag*, *p* < 0.001; *Opalin*, *p* < 0.01; *Pmp22*, *p* < 0.001; *Sox10*, *p* < 0.01); oligodendrocyte differentiation (*Opalin*, *p* < 0.01; *Gjb1*, *p* < 0.01); microglial function (*Kcnk13*, *p* < 0.01); DNA damage (*Hus1*, *p* < 0.01; and *Pttg1*, 0.01); immune cell recruitment/activation (*Lamp1*, *p* < 0.001 and *Gpr183*, *p* = 0.001); and inflammation (*Gpr183*, *p* = 0.001) (Figure 4A). ES mice also showed an up-regulation of genes associated with the clearance of dead cells and debris (*Mertk*, *p* < 0.01 and *Atg14*, *p* < 0.01); proper protein folding (*Hspb1*, *p* = 0.001); and the regulation of cell growth (*Cdkn1a*, *p* = 0.001) in response to stress (Figure 4A). Compared to HC, IS showed a down-regulation of genes associated with DNA repair and protection from neurodegeneration (*Mre11a*, *p* < 0.01; *ErCC2*, *p* = 0.001; *Fen1*, *p* < 0.01); microglial function (*Kcnk13*, *p* < 0.01); cell survival and differentiation (*Pik3r2*, *p* < 0.01); and blood–brain barrier (BBB) protection (*CD44*, *p* < 0.01) (Figure 4B). IS showed an up-regulation of genes involved in cytokine signaling (*Tnfrsf25*, *p* = 0.01); activation of the innate immune response (*Lcn2*, *p* = 0.01); recruitment of neutrophils (*Lcn2*, *p* = 0.01 and *Ncf1*, *p* = 0.01); and the *Arc* gene (Figure 4B).

Analysis of gene expression via relative mRNA levels within HPC between ES and IS groups revealed significantly different expression levels. Compared to ES, IS showed a down-regulation of genes associated with protection from neurodegeneration (*Cdk20*, *p* < 0.01 and *Mre11a*, *p* < 0.01); There was an up-regulation of genes involved in the pro-inflammatory response (*Traf6*, *p* < 0.01); genes associated with DNA damage (*Pttg1*, *p* < 0.01); the recruitment, differentiation, and migration of inflammatory cells (*Traf6*, *p* < 0.01; *Itga7*, *p* < 0.01; *Kcnk13*, *p* < 0.01); increased microglia activity (*Kcnk13*, *p* < 0.01); and remyelination (*Pmp22*, *p* < 0.01; *Opalin*, *p* = 0.01; *Mobp*, *p* = 0.01; *Kmt2c*, *p* = 0.01) (Supplementary Figure S3A).

### 3.5. Neuroinflammation Related to Neurodegeneration

Analyses of gene markers of neuroinflammation and inflammatory-associated pathways in the BLA indicated significant differences between groups. Furthermore, neurodegenerative-related pathways also showed significant differences between groups. Overall, following CTX, ES showed increases in neuroprotective markers following CTX while IS showed increases in markers of immune cell recruitment and activation, and neuron repair. The MT control group showed up-regulation of genes associated with glucose transportation (*Slc2a1*, *p* < 0.001); microglial function (Dido1, *p* < 0.01); the maintenance of the neuromuscular junction (*Lrp4*, *p* < 0.01); transmitter uptake and release (*Igf1r*, *p* = 0.01; *Tspo*, *p* = 0.01); vesicle trafficking (*Sv2a*, *p* < 0.05); and cytokine signaling (*Tgfbr1*, *p* < 0.05) compared to HC (Supplementary Figure S2B).

**Figure 5:**
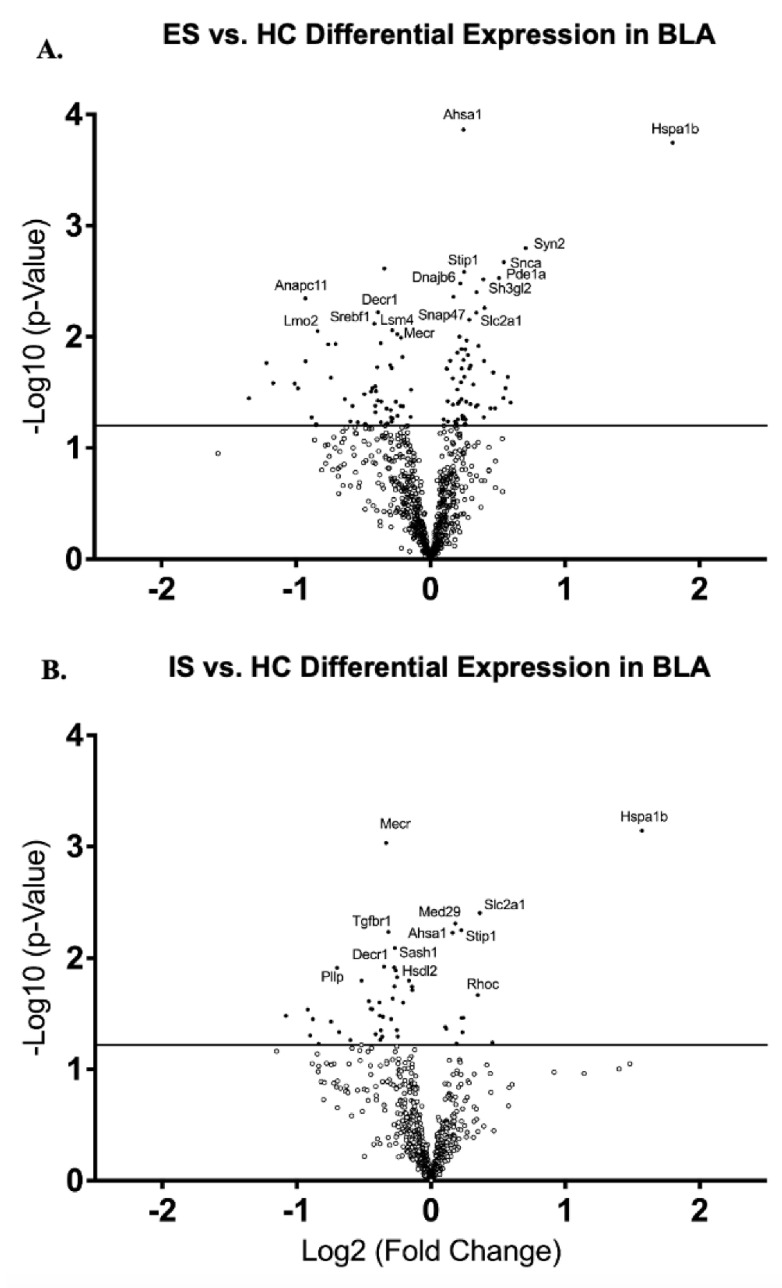
**Escapable Stress Increases the Expression of Neuroprotective Genes.** Volcano plot displaying each gene expression levels in BLA compared to home cage (HC) control following CTX for (**A**) escapable stress (ES) and (**B**) inescapable stress (IS) groups. Statistically significant genes fall above the horizontal line, and highly differentially expressed genes fall to either side of the zero on the *x*-axis. The most relevant genes are labeled in the plot.

Analysis of gene expression via relative mRNA levels within BLA revealed significantly different expression levels between ES and IS groups when compared to HC. ES mice showed a down-regulation of many genes associated with the mediation of protein degeneration (*Anapc11*, *p* < 0.01); fatty acid synthesis and metabolism (*Srebf1*, *p* < 0.01; *Mecr*, *p* < 0.01; and *Decr1*, *p* < 0.01); pre-mRNA splicing (*Lsm4*, *p* < 0.01); and stem cell development (*Lmo2*, *p* < 0.01) (Figure 5A). ES mice also showed an up-regulation of genes involved in synaptogenesis (*Syn2*, *p* = 0.001); neurotransmitter release (*Syn2*, *p* = 0.001); vesicle trafficking and budding (*Snca*, *p* < 0.01 and *Sh3g12*, *p* < 0.01); signal transduction (*Pde1a*, *p* < 0.01); the suppression of protein aggregation and nerve damage (*Dnajb6*, *p* < 0.01 and *Snap47*, *p* < 0.01); proper protein folding (*Hspa1b*, *p* < 0.001 and *Stip1*, *p* < 0.01); glucose transportation (*Slc2a1*, *p* < 0.01); and T-cell signaling (*Ahsa1*, *p* = 0.0001) (Figure 5A). IS mice showed a down-regulation of genes associated with signal transduction (*Tgfbr1*, *p* < 0.01; cytokine production (*Sash1*, *p* < 0.01); myelination (*Pllp*, *p* = 0.01); and fatty acid synthesis and metabolism (*Mecr*, *p* < 0.001; and *Decr1*, *p* = 0.01) (Figure 5B). There was an up-regulation of genes involved in T-cell signaling (*Ahsa1*, *p* < 0.01); proper protein folding (*Hspa1b*, *p* < 0.001 and *Stip1*, *p* < 0.01); and glucose transportation (*Slc2a1*, *p* < 0.01) (Figure 5B).

Analysis of gene expression via relative mRNA levels within BLA between ES and IS groups revealed significantly different expression levels. Compared to ES, IS showed a down-regulation of genes associated with signal transduction and synaptic transmission (*Pde1a*, *p* < 0.001 and *Synj1*, *p* < 0.01), vesicle trafficking and budding (*Sh3g12*, *p* < 0.001; *Nsf*, *p* < 0.001; *Snca*, *p* < 0.01; Rab3c, *p* < 0.01), and protein interaction and maintaining protein homeostasis (*Psmc6*, *p* < 0.01 and *Wac*, *p* < 0.01) (Supplementary Figure S3B). 

### 3.6. Regional Pathway Regulation

Pathway regulation scores were determined using the nSolver database using directed global significance scores of overlaid differential gene expression data for sets of genes grouped by biological function relative to HC. This analysis measures the extent to which genes within a given set are up- or down-regulated with the independent variable.

**Figure 6:**
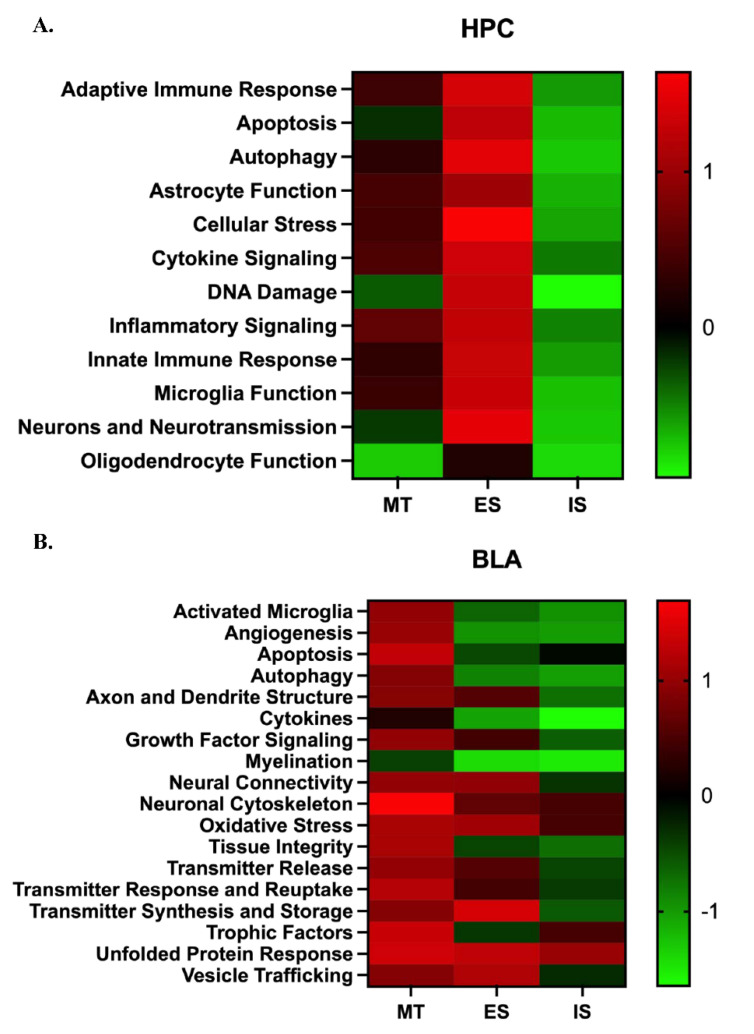
**Stressor Controllability Differentially Influences Pathways Involved in Brain Homeostasis.** Heatmaps displaying the directed global significance scores of overlayed differential gene expression data for sets of genes grouped by biological function relative to HC in (**A**) HPC and (**B**) BLA, respectively. Directed global significance statistics measure the extent to which a gene set’s genes are up- or down-regulated with the variable. Red denotes gene sets whose genes exhibit extensive over-expression with the covariate, green denotes gene sets with extensive under-expression.

Data within HPC revealed differences between ES and IS groups when compared to HC. ES mice showed an overall up-regulation in immune-related pathways while IS mice showed an overall down-regulation. MT did not show any major differences in pathway regulation scores compared to HC (Figure 6A). Analysis of pathway regulation scores based on differential gene expression data within BLA revealed differences between the ES and IS groups when compared to HC. ES mice showed an overall up-regulation in pathways related to neuronal connectivity and neurotransmitter/vesicle trafficking. IS mice showed an overall down-regulation in all pathways with the exception of oxidative stress, trophic factors, and the unfolded protein response. MT mice showed an up-regulation in all pathways apart from cytokines and myelination compared to HC (Figure 6B).

## 4. Discussion

In the current study, we assessed the effects of fear memory recall associated with ES and IS on neuroinflammatory processes in BLA and HPC, and their relationships to alterations in sleep, behavioral fear, and the stress response as indicated by SIH. Given that only minimal differences between HC and MT control groups were found within the gene expression data, and no differences were found for any other parameter examined, we chose to focus our efforts on differences found between the ES and IS groups. Supporting previous findings, our results showed subsequent REM responses directionally differ in response to controllable (ES) and uncontrollable (IS) stress, and after CTX associated with ES and IS [25,54], and also differences in NREM sleep within the dark period following ST. Currently, it is unknown how fear recall associated with ES and IS after CTX influences the neuroimmune response and its relationship to sleep. Results of our study demonstrate for the first time that, within HPC and BLA, memories associated with ES down-regulates many genes associated with neuroinflammation and an up-regulates genes associated with neuroprotection whereas memories associated with IS down-regulates genes associated with neuroprotection and up-regulates genes involved in neuroinflammation. Additionally, the changes in neuroimmune responses were directly associated with changes in sleep, establishing a bidirectional relationship between the two systems and their potentially important role in mediating stress outcomes. In contrast, freezing and SIH did not differ regardless of whether the stress was controllable or not.

The role of stress in the connection between sleep quality and immunological processes has been confirmed in a number of reports [20,45,58,59,60], with the type, duration, and intensity of the stressor being important in influencing responses [17,61]. Specifically, stressor controllability has been demonstrated to produce directionally different outcomes in sleep [25,54] and immune responses [53]. A number of studies indicate that increased levels of pro-inflammatory cytokines play an important role in the etiology of fear- and anxiety-based psychiatric disorders [62,63] and the association between sleep disturbances and neuroinflammation seems to have a key impact on the development and course of various fear- and anxiety-based psychiatric disorders [46,47,59,60,64,65,66,67]. Our study illustrates that the persistent resurfacing of fear memories may provide a pathway for long-term influences of stress on sleep and inflammatory signaling, which may be impacted by how controllability alters the formation of those memories.

Maladaptive changes in fear neurocircuitry may be central to mediating pathological fear memory. Specifically, the amygdala is important for the formation and processing of fear memories, fear learning, and the manifestation of fear behaviors [2,68,69,70,71]. The HPC has reciprocal connections to the amygdala and is responsible for the consolidation and long-term storage of fear memories [68,71,72,73,74]. The HPC forms contextual associations with fearful stimuli and takes part in the retrieval of fear memories from the amygdala [71,73,74,75,76,77], and determines if a fearful context is threatening or non-threatening by regulating HPA activity [74,78]. Furthermore, the amygdala sends projections to brainstem regions necessary for the expression of fear behaviors and the regulation of sleep/arousal states [26,79,80]. Firing rates of pyramidal cells increase significantly during REM compared to other sleep/wake states, indicating a strong connectedness between the amygdala and HPC during REM [34]. The HPC also has involvement in stress-induced changes in sleep by regulating REM theta wave synchrony between itself and the amygdala [81,82,83]. The theta frequency band is important for memory formation and integration [81,84], and it is believed that theta rhythms are key to hippocampal memory consolidation [81,82]. Thus, connections between the amygdala and HPC may be critical for mediating the effects of stress and fear memories on sleep [34,82,85].

### 4.1. Stress, Fear Memory, and Sleep

REM is thought to be important in fear memory consolidation [31,37,86,87] and emotional processing [26,31,33,37,86,87,88,89,90]. Yet, our experiments show no evidence that supports the hypothesis that REM is necessary for the formation or consolidation of fear memories, but do indicate that controllability may alter the effects of fear and fear memory across multiple systems. We demonstrated that ES not only prevents a loss of REM but increases the total amount of REM following ST throughout the entirety of the sleep period. These findings agree with previous studies which demonstrate stressor controllability differentially alters subsequent REM sleep responses following ST and CTX [25,54].

Controllability also differentially altered total NREM sleep exclusively within the dark period, providing evidence that NREM sleep may also play a role in regulating stress-induced alterations in the immune system [45,59,91,92,93,94]. Levels of circulating cytokines and inflammatory proteins have been shown to fluctuate with circadian rhythms [41,93,95], and circadian misalignment can alter this fluctuation [41]. Interestingly, the increase in NREM we found in B6 mice was not found in previous studies conducted in BALB/c mice [25,54], suggesting strain differences in the effects of controllable and uncontrollable stress on sleep. These strains show several differences in responses to stress, including differences in prolactin [96] and differences in the relative REM response to IS [97], that are consistent with this suggestion.

Given that there was no difference in behavioral fear (freezing) but different alterations in sleep following stress exposure, these data suggest that sleep may have a role in processing emotional memories, potentially determining whether or not they lead to psychopathology. The increase in sleep after training with controllable stress, and memories associated with it after CTX, suggests it may play a positive role in the processing of fearful emotion and memories that is consistent with other suggestions regarding sleep and the adaptive processing of emotion [26,31,33,37,86,87,88,89,90] that might also involve differences in neuroinflammation.

### 4.2. Stress and Neuroinflammation

The immune system and the central nervous system (CNS) have bidirectional communication [17,20,98,99,100] that is mediated by the stress response [17,20,101,102]. One reported consequence of stress exposure is a pro-inflammatory immune response within the brain [17,18,101,102]. Inflammatory responses by immune cells are mediated by various intrinsic and extrinsic factors, including cytokines, neuropeptides, and stress hormones [17,103,104,105,106]. The effects of various types of stressors on the brain’s immune system have been debated. However, prior work has demonstrated that controllability can differentially alter the immune response [54], with uncontrollable stress contributing to immune system dysfunction and controllable stress suppressing many indices of neuroinflammation [20].

Stress and sleep loss have also been shown to induce neuroinflammation [17,19,20,43,45,107]. Furthermore, immune signaling can influence behavior [108]. However, the majority of studies have measured aspects of immunity during stress in an isolated manner [109,110,111,112,113]. Studies attempting to correlate stress with immunity and sleep are limited, and we are unaware of prior studies which compared neuroimmune and sleep responses elicited by fearful memory. It has been determined that stress-induced sleep loss can provoke alterations in immune system function [17,47,59,114], indicating strong connections between the stress and sleep neural circuitry and the immune system.

### 4.3. Clinical Implications

The immune system plays a role in the pathologies of various neuropsychiatric illnesses [64,115]. Stress can directly alter gene expression, influencing the immune response. This can lead to significant damage, distorting neuronal signaling, and disrupting normal brain function [107]. This has direct implications for fear- and anxiety-based psychiatric disorders, and may serve as an early indicator for the development of neurodegenerative diseases, as patients with these disorders all show increased neuroinflammation [64,106,108,115,116]. The HPC and BLA have implicated involvement in fear- and anxiety-based disorders, including PTSD [111]. Recently, studies have shown individuals diagnosed with these disorders are more likely to develop neurodegenerative diseases later in life [46,64,66,106,115,116,117,118], and BLA atrophy is associated with early AD [119]. Thus, understanding potential early effects of stress may provide insight into its long-term effects on AD-related neuroimmune pathways.

Our results indicate that fear memories associated with controllable stress can result in increases in sleep and less neuroinflammation whereas those associated with uncontrollable stress can result in decreased sleep and greater neuroinflammation. Furthermore, differences in the regulation of pathways related to immune responsivity and brain homeostasis suggest that stressor controllability, or its perception, is a key factor in determining the type of neuroimmune response. Together, these data indicate that fear memories have the ability to elicit different sleep and immune responses dependent upon whether the initial stressor associated with their formation was controllable or not in ways that have relevance to the development of psychopathologies, and potentially neurodegeneration. However, the current study does not determine whether fear memory-induced neuroinflammation causes sleep loss or if stress-induced sleep loss promotes neuroinflammation. It is plausible, even likely, that fearful emotion associated with controllable and uncontrollable stress, and respective fear memories, drives both sleep and neuroinflammatory responses. It will thus be important to determine whether differential activation of fear circuitry by ES and IS regulates fear conditioned sleep and neuroinflammatory responses, and whether they are co-regulated.

### 4.4. Study Limitations

One potential concern for our results relates to the use of isoflurane for sedation prior to tissue collection at the conclusion of the study. Previous findings have shown that chronic isoflurane exposure can have a lasting effect on gene expression [120]. However, other studies show differential effects in gene expression levels in acute versus chronic isoflurane exposure, where chronic exposure showed increased evidence of damaging effects in the brain [121], and that isoflurane did not stop ongoing transcription but did prevent the initiation of new transcription [122]. The 2 h delay in euthanizing the mice was sufficient time for normal transcription rates to occur, and the brief exposure to isoflurane the mice received should not have altered fear memory induced transcription that occurred prior to euthanasia.

## 5. Conclusions

Our study found that stressor controllability differentially influences sleep and neuroimmune responses in a directionally different manner, even though major indicators of stress and behavioral fear are virtually identical. Specifically, the ability to control initial stress results in increased sleep following fear memory recall as described previously, and this increase was associated with protective effects in the immune response as hypothesized. The differential sleep and neuroimmune responses appear to be integrated during fear conditioning (ST) and then reproduced by fear memory recall (CTX). The established roles of disrupted sleep and neuroinflammation in stress-related disorders indicate that these differences may serve as informative indices of how fear memory can lead to psychopathology. The ES and IS paradigm we used provides a model for assessing how fear memory impacts sleep and neuroinflammation at the mechanistic level.

## Figures and Tables

**Table 1:** Total duration (in min) of REM sleep ± SEM for each experimental day across treatment groups.

	HC	MT	ES	IS
**Base (20 h)**	46.8 ± 6.7	40.2 ± 7.6	45.1 ± 7.0	48.7 ± 5.9
**ST1 (20 h)**	42.9 ± 3.8 ^	45.3 ± 7.5 +	54 ± 6.9 *	30.2 ± 10.7
**ST2 (20 h)**	44.6 ± 15.6 ^	45.9 ± 3.5 +	58.8 ± 7.9 *	30.8 ± 9.3
**CTX (2 h)**	11.0 ± 0.9 ^	6.4 ± 1.4 +	5.6 ± 0.5 *	4.8 ± 0.7

Significant differences in HC vs. IS: ^ *p* < 0.05. Significant differences in MT vs. IS: + *p* < 0.05. Significant differences in ES vs. IS: * *p* < 0.05. Recording time for each day is indicated in parentheses.

**Table 2:** Total duration (in min) of NREM sleep ± SEM for each experimental day across treatment groups.

	HC	MT	ES	IS
**Base (20 h)**	506.8 ± 14.5	526.8 ± 6.7	535.6 ± 21.9	512.8 ± 21.3
**ST1 (20 h)**	533.9 ± 24.5	532.0 ± 15.3	600.5 ± 31.5 *	524.3 ± 11.9
**ST2 (20 h)**	538.3 ± 18.4	549.6 ± 30.5	570.3 ± 23.5 *	522.5 ± 23.7
**CTX (2 h)**	76.5 ± 5.9	79.5 ± 3.9	61.9 ± 3.3	70.4 ± 4.2

Significant differences in ES vs. ISL * *p* < 0.05. Recording time for each day is indicated in parentheses.

## Data Availability

Experimental data available upon request.

## Supplementary Materials

The following are available online at https://www.mdpi.com/article/10.3390/life12091320/s1, Figure S1: Sleep Does Not Differ Between Home Cage and Mock Trained Control Groups. Total REM duration following Baseline (Base), shock training days (ST 1 and ST 2), and context re-exposure (CTX) during the (A) first 2 h, (B) total 20 h. Total NREM duration following Base, ST 1 and ST 2, and CTX during the (C) first 2 h, (D) total 20 h; Figure S2: Gene Expression Does Not Differ Greatly Between HC and MT. Volcano plot displaying gene expression levels in (A) HPC for MT compared to HC and (B) BLA for MT compared to HC. Statistically significant genes fall above the horizontal line, and highly differentially expressed genes fall to either side of the zero on the x-axis. The most relevant genes are labeled in the plot; Figure S3: Inescapable Stress Increases the Expression of Pro-inflammatory Immune Genes and Decreases the Expression of Neuroprotective Genes Compared to Escapable Stress. Volcano plot displaying each gene expression levels in (A) HPC for IS compared to ES following CTX and (B) BLA for IS compared to ES following CTX. Statistically significant genes fall above the horizontal line, and highly differentially expressed genes fall to either side of the zero on the x-axis. The most relevant genes are labeled in the plot; Table S1: REM sleep as a percentage of baseline for each experimental day across treatment groups. Recording time for each day is indicated in parentheses.
