# Supplementary material for: Controllable and Uncontrollable Stress Differentially Impact Fear Conditioned Alterations in Sleep and Neuroimmune Signaling in Mice
Source: Life (Basel). 2022 Aug 26;12(9):1320. doi: 10.3390/life12091320 (PMC9506236; doi:10.3390/life12091320)
Supplement: Supplementary file 1 [file life-12-01320-s001.zip › life-1804753-supplementary.pdf]

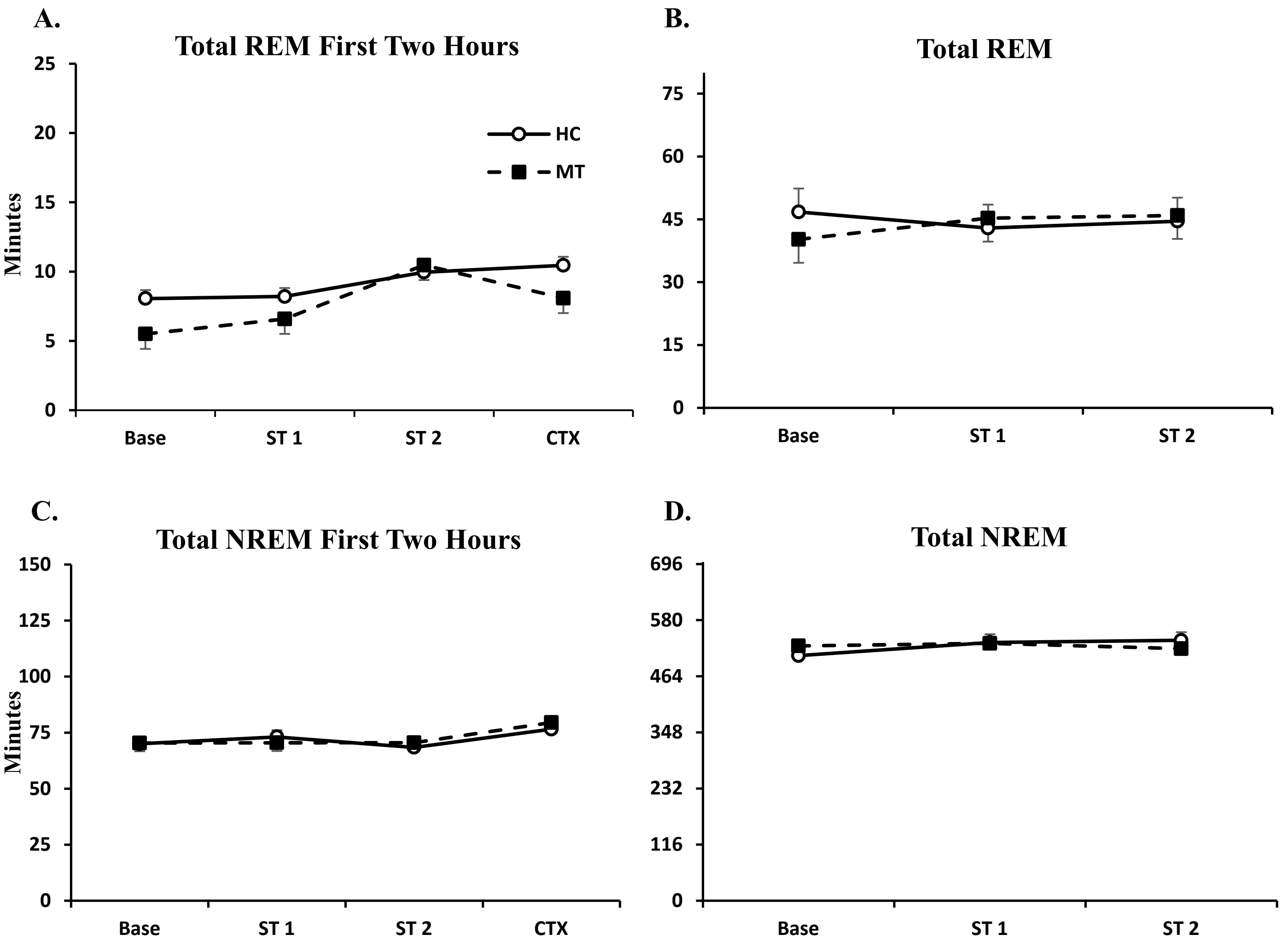

Supplementary Figure S1. Sleep Does Not Differ Between Home Cage and Mock Trained Control Groups. Total REM duration following Baseline (Base), shock training days (ST 1 and ST 2), and context re-exposure (CTX) during the (**A**) first 2 h, (**B**) total 20h. Total NREM duration following Base, ST 1 and ST 2, and CTX during the (**C**) first 2h, (**D**) total 20h.

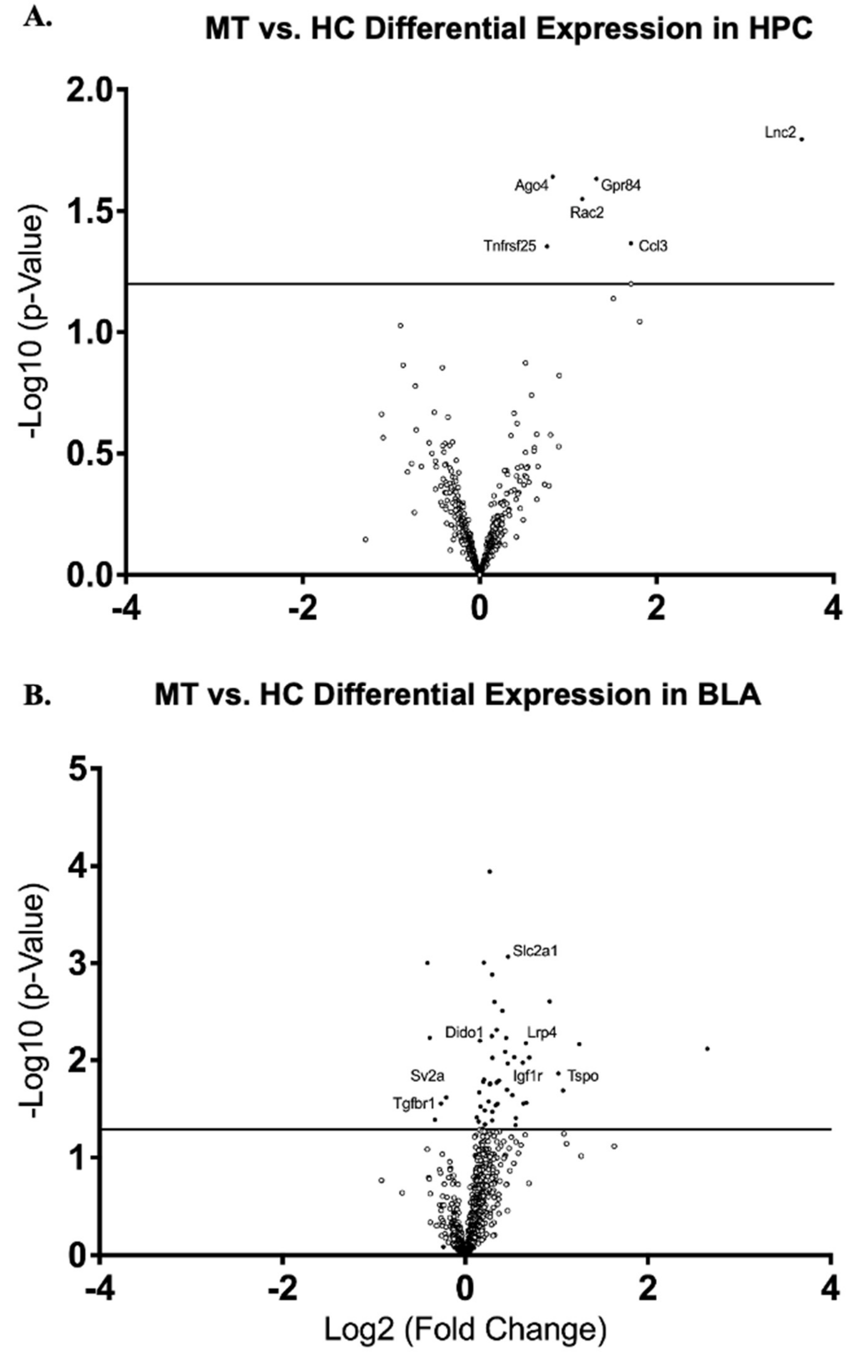

Supplementary Figure S2. Gene Expression Does Not Differ Greatly Between HC and MT. Volcano plot displaying gene expression levels in (**A**) HPC for MT compared to HC and (**B**) BLA for MT compared to HC. Statistically significant genes fall above the horizontal line, and highly differentially expressed genes fall to either side of the zero on the x-axis. The most relevant genes are labeled in the plot.

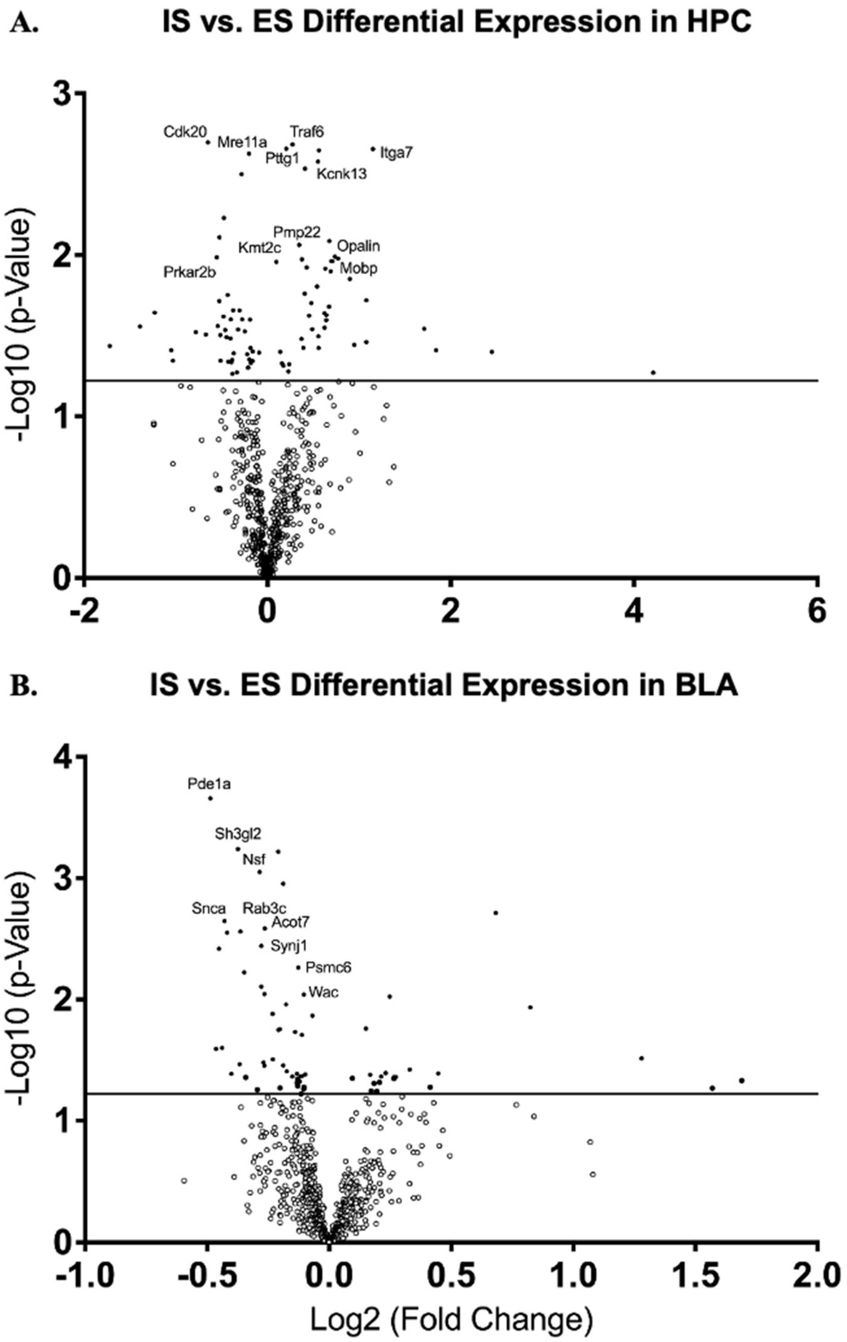

Supplementary Figure S3. Inescapable Stress Increases the Expression of Pro-inflammatory Immune Genes and Decreases the Expression of Neuroprotective Genes Compared to Escapable Stress. Volcano plot displaying each gene expression levels in (**A**) HPC for IS compared to ES following CTX and (**B**) BLA for IS compared to ES following CTX. Statistically significant genes fall above the horizontal line, and highly differentially expressed genes fall to either side of the zero on the x-axis. The most relevant genes are labeled in the plot.

Supplementary Table S1. REM sleep as a percentage of baseline for each experimental day across treatment groups. Recording time for each day is indicated in parentheses.

	**HC**	**MT**	**ES**	**IS**
**ST1 (20h)**	7.75	7.99	9.30	5.38
**ST2 (20h)**	8.06	8.10	10.13	5.49
**CTX (2h)**	12.57	7.45	8.30	6.38
